# Cross sectional study of chronic hepatitis B prevalence among healthcare workers in an urban setting, Sierra Leone

**DOI:** 10.1371/journal.pone.0201820

**Published:** 2018-08-10

**Authors:** Thomas A. Massaquoi, Rachael M. Burke, Guang Yang, Suliaman Lakoh, Stephen Sevalie, Bo Li, Hongjun Jia, Lei Huang, Gibrilla F. Deen, Fenella Beynon, Foday Sahr

**Affiliations:** 1 34 Military Hospital, Wilberforce Barracks, Freetown, Sierra Leone; 2 Centre for Global Health and Health Partnerships, Faculty of Life Sciences & Medicine, King’s College London, London, United Kingdom; 3 Kings Sierra Leone Partnership, Connaught Hospital, Freetown, Sierra Leone; 4 Chinese Military Medical Expert Group in Sierra Leone, 302 Military Hospital, Beijing, China; 5 University Sierra Leone Teaching Hospital Complex, Connaught Hospital, Freetown, Sierra Leone; University of Cincinnati College of Medicine, UNITED STATES

## Abstract

**Introduction:**

Hepatitis B is a serious public health problem across sub-Saharan Africa. Sierra Leone has no national hepatitis B strategy plan or high quality estimates of prevalence. Healthcare workers are perceived as an at-risk group for hepatitis B. We assessed the prevalence of hepatitis B among healthcare workers at two hospital sites in Freetown, Sierra Leone.

**Methods:**

In October 2017, healthcare workers were offered voluntary testing for hepatitis B surface antigen (HBsAg), hepatitis B surface antibody (anti-HBs), hepatitis B core antibody (anti-HBc), hepatitis B e antigen (HBeAg) and hepatitis B e antibody (anti-HBe) using rapid lateral flow assay for all samples, followed by Enzyme Immunosorbent Assay to confirm positive results. Participants completed a questionnaire about knowledge, attitudes and practices concerning hepatitis B. HBsAg positive participants were invited to a clinic for further assessment.

**Results:**

Overall, 447 participants were tested for hepatitis B. Most (90.6%, 405/447) participants were nurses, 72.3% (323/447) were female and 71.6% (320/447) were 30 years or older. The prevalence of chronic hepatitis B (HBsAg positivity) was 8.7% (39 / 447, 95% CI 6.3–11.7%). There was no significant difference in prevalence by sex, age group, site of work or type of job. None of the 66.7% (26 / 39) of participants with chronic hepatitis B who attended the clinic met the 2015 WHO criteria to start treatment for hepatitis B on the basis of cirrhosis. Most participants (96.9% 432 / 446) stated that they were worried about their risk of hepatitis B at work.

**Conclusions:**

Hepatitis B is highly prevalent among healthcare workers in Sierra Leone. It is unclear whether this reflects high community prevalence or is due to occupational risk. No participants with chronic hepatitis B needed to start treatment. In order to achieve the WHO target of elimination of viral hepatitis by 2030, introduction of birth dose vaccine for infants and catch-up vaccines for healthcare workers and healthcare students, together with a national hepatitis B screen and treat programme is advisable for Sierra Leone.

## Introduction

Hepatitis B is a serious and growing public health problem. Worldwide, viral hepatitis caused 1.45 million deaths in 2013, which represents an increased mortality burden by 63% between 1990 and 2013 [1]. The leading cause of viral hepatitis in sub-Saharan Africa is hepatitis B [1]. The World Health Organisation (WHO) estimates the prevalence of chronic hepatitis B infection in sub-Saharan Africa is 6.1% [2]. This is a substantial proportion of people at risk of complications including cirrhosis, hepatocellular carcinoma and premature mortality. The WHO have set a target of elimination of viral hepatitis by 2030, and substantial public health effort will be required to reach this laudable aim. Whilst hepatitis B is vaccine-preventable and many countries have introduced vaccinations in the past 20 years, most countries in sub-Saharan Africa have hepatitis B vaccines for infants only without an adult catch up plan and coverage is imperfect– 74% of eligible children in sub-Saharan Africa received three doses of hepatitis B vaccine in 2016 [3]. The WHO published the first set of guidelines for assessment and treatment of people with chronic hepatitis B in 2015 [4]. Nucleoside analogues to treat hepatitis B are widely available to purchase privately, however the cost of treatment to individuals in low-income countries is often prohibitive and few countries in sub-Saharan Africa provide publically funded treatment for hepatitis B mono-infection [5] [6]. Healthcare workers are perceived to be a high-risk group for hepatitis B due to occupational exposure to infected bodily fluids and often poor availability of protective equipment in sub-Saharan Africa [7] [8].

There is no national strategy plan for prevention and control of hepatitis B in Sierra Leone either among healthcare workers or the general population, and no routine surveillance or sero-surveys of infection [9]. Infant hepatitis B vaccination has been available in Sierra Leone since 2007 as part of Expanded Programme of Immunisation, although coverage of three does is only 84% [3]. Birth-dose vaccination (which is an essential part of preventing mother to child transmission) has yet to be introduced and there are no free or subsidised vaccines available for adults in general or healthcare workers in particular. Nucleoside analogue treatment for hepatitis B mono-infection is available in Sierra Leone, but only through private clinics and requiring substantial out-of-pocket payment. Treatment for hepatitis B and HIV co-infection is provided for free through the National HIV/AIDS Control Programme.

The present study was undertaken to provide evidence about the prevalence of serological markers of Hepatitis B virus (HBV) exposure, infection and vaccination among healthcare workers in Sierra Leone, and observe the proportion of HBsAg-positive people with cirrhosis and other complications of hepatitis B infection. We also intended to assess healthcare worker knowledge, attitudes and practices regarding hepatitis B. This is in order to inform national public health policy planning regarding hepatitis B in Sierra Leone, which is essential in order to contribute to meeting the WHO goal for elimination of viral hepatitis B by 2030.

## Methods

This study was conducted in October 2017 among staff at a tertiary teaching hospital (Connaught Government Hospital) and a military-run general hospital (34 Military), both located in Freetown, the capital city of Sierra Leone. Up to 605 doctors and nurses are registered to work at Connaught Government Hospital, but this includes individuals not working during the study period e.g. those on long-term study leave. Two hundred and forty doctors and nurses worked at 34 Military Hospital during the study period, these include both military and civilian workers.

Following a period of sensitisation and explanation about the study among staff; voluntary hepatitis B testing was offered to all doctors and nurses working at these two sites. Over a four week period, a team of research nurses went to every hospital ward at least once during each nursing shift and invited nurses to come for voluntary hepatitis B testing. Doctors were informed about the study in person and via electronic messaging. The study team collected information on numbers of potential participants (i.e. nurses who were present at work that day) invited during ward mobilisation. Other healthcare workers who heard about the study from colleagues and attended the study room were also allowed to enrol even if they had not been present whilst the study nurses had visited their ward.

Pre-test counselling was conducted by trained research nurses in a dedicated private room on each hospital site, and written informed consent was obtained. Participants were asked to complete a short questionnaire using an offline electronic form on a tablet computer using EpiInfo companion for Android (CDC Atlanta, GA). This questionnaire consisted of nine “yes / no” questions about knowledge, attitudes and practices, and five “yes / no / don’t know” questions about knowledge of routes of transmission of hepatitis B.

Peripheral blood samples (5mL volume) were collected by study lab technicians into a serum separator tube (BD Vacutainer SST advance). Whole blood samples were stored at room temperature (25°C) for a maximum of 6 hours, then separated by centrifugation at 2000 rpm for 10 minutes before storage at 4°C for a maximum of 36 hours prior to testing by Lateral Flow Assay (LFA). Serological testing for hepatitis B surface antigen (HBsAg), hepatitis B surface antibody (anti-HBs), hepatitis B core antibody (anti-HBc), hepatitis B e antigen (HBeAg) and hepatitis B e antibody (anti-HBe) were performed on serum using a KHB Multi-HBV Markers Colloidal Gold LFA (Shanghai Kehua Bio-Engineering, Shanghai, China) according to the manufacturers instructions under laboratory conditions. All samples positive for HBsAg, and those with isolated anti-HBc were stored in the serum separator tubes at 4°C for a maximum of two weeks before batch Enzyme Immunosorbent Assay (EIA) hepatitis B serology panel testing for confirmation using Wantai Bio-Pharm EIA kits (Beijing Wantai Biological Pharmacy, Beijing, China). The manufacturer reported specificity, sensitivity and limits of detection (LOD) for all LFA and EIA tests are listed in S1 Appendix. Note that the anti-HBs reported LOD for LFA is 30 IU/mL. HBsAg sensitivity is 99.1% with 5 IU/mL LOD by KHB LFA and 100% with 1 IU/mL LOD by Wantai EIA. Despite the higher sensitivity, cost constraints meant that we could not perform EIA testing on all negative samples. The KHB LFA tests are widely used in China and the Wantai EIA test has been used in previous studies [10]. Testing was conducted in the Chinese Military Medical Expert laboratory within the 34 Military Hospital site.

Results were given back to patients individually in sealed envelopes, with written information about interpretation of test results. Research nurses were available to explain results and answer queries should a participant wish. Participants who tested positive for HBsAg were invited to a free medical consultation including liver function and platelet count testing to evaluate clinical sequelae of their hepatitis B infection and assess whether they met criteria for treatment based on 2015 WHO guidelines [4]. Abdominal ultrasound and hepatic elastography measurements were not available. Those who did not attend clinic initially were contacted by phone to encourage them to attend for blood tests and medical consultation. All participants with HBsAg or anti-HBc were advised that they should have an HIV test and given information about how to access HIV testing. HIV test results were only collected for people who attended the follow up clinic. Our protocol stated that participants with chronic hepatitis B without co-existent HIV, who met 2015 WHO criteria for treatment would be counselled about their disease and advised to pay for tenofovir treatment, but no-one in the study met these criteria. Those who did not meet criteria for treatment were advised to monitor liver function, platelets and abdominal ultrasound (where available) annually or more frequently if liver function was deranged (defined as alanine transaminase (ALT) >19 in women or >30 men), in keeping with WHO guidance [4].

Data was entered into an secure EpiInfo (CDC Atlanta, GA) database and descriptive analysis was conducted in stata 15 (StataCorp, College Station, TX).

This study was approved by the Sierra Leone Ethics and Scientific Review Committee.

## Results

In total, 447 participants took part in the study; 301 from Connaught Government Hospital and 146 from 34 Military Hospital.

**Fig 1:**
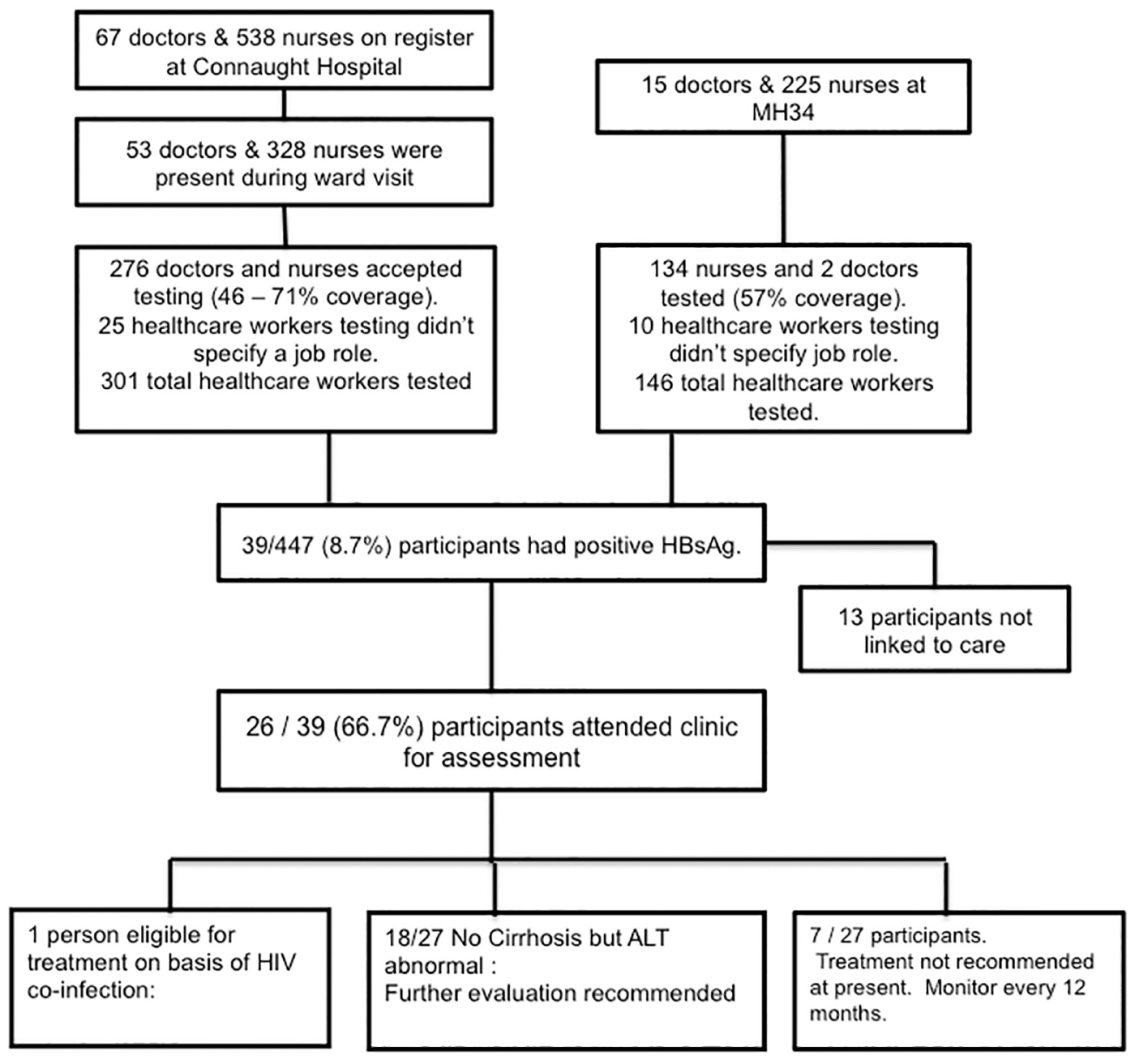
Flow chart of recruited participants. Numbers of potential participants at Connaught Government Hospital and 34 Military Hospital, number recruited to study, and number HBsAg positive participants linked to care.

Whilst up to 605 doctors and nurses work at Connaught Government Hospital, 381 were identified as at work during ward mobilisation visits during the study period. It is possible that some of the participants were not among those present at the time of a ward mobilisation visits, but heard about the study from colleagues. Accordingly, the response rate for doctors and nurses was between 45.6% (276 / 605) and 72.4% (276 / 381). At 34 Military Hospital, 240 doctors and nurses were invited to participate, and the response rate was 56.7% (136 / 240). A further 35 healthcare workers (25 at Connaught and 10 at 34 Military) participated in the study but did not indicate their job category. Fig 1 shows the flow chart of recruited participants. Overall, 72.3% (323 / 447) of participants were female and 90.6% (405/447) were nurses.

HBsAg was positive in 8.7% (39/447) (95% CI: 6.3–11.7%) using LFA with all positives confimed on EIA (Table 1. Of the HBsAg positives, all were HBeAg negative and 87.2% (34/39) were anti-HBe positive.

**Table 1:** Demographics of recruited participants and those with chronic hepatitis B.

	All participants, N	HbsAg positive, N(%)
N =	447	39 (8.7%)
Location		
34 Military Hospital	146	13 (8.9%)
Connaught Hospital	301	26 (8.6%)
Sex		
Male	124	8 (6.4%)
Female	323	31 (9.6%)
Age group		
Under 30	127	9 (7.1%)
30 or over	320	30 (9.4%)
Cadre		
Nurse	405	36 (8.9%)
Doctor	7	0 (0%)
Unknown or didn’t answer question	35	3 (8.6%)

Among HBsAg negative samples, 21.9% (7/406) samples were positive for both anti-HBc and anti-HBs, and 20.2% (82 /406) positive for anti-HBc alone by LFA testing. When re-tested by EIA, 40.7% (33/81) of these initially “anti-HBc alone” samples had anti-HBs detected (one sample had too small a blood volume for EIA testing). Two participants who had anti-HBe only on LFA were recalled for re-testing by EIA (their blood samples had not originally been stored). One had both anti-HBe and anti-HBc on EIA, and the other could not re-attend. Sixteen samples were positive for anti-HBs only (Table 2).

**Table 2:** HBV serology results.

	After initial LFA testing (N = 447)	After LFA plus EIA (N = 447, 120 samples re-tested)
Chronic infection (HBsAg positive)	39 (8.7%)	39 (8.7%)
HBsAg + HBeAg	0	0
HBsAg + anti-HBe	34	33
HBsAg without anti-HBe or HBeAg	5	6
Exposed (anti-HBc positive without HBsAg)	89 (19.9%)	90 (20.1%)
Anti-HBc and anti-HBs	7	40
Anti-HBc alone	82	50
Consistent with immunisation (anti-HBs without anti-HBc)	16 (3.5%)	16 (3.5%)
Anti-HBe only	2	1 *
Negative (no hepatitis antigens or antibodies detected)	301 (67%)	301 (67%)

* Two patients with anti-HBe alone were recalled for repeat sampling, one attended and had anti-HBc detected on EIA, the other did not return.

None of the 66.7% (26 / 39) HBsAg positive participants who attended hepatitis clinic for evaluation had any clinical evidence of decompensated cirrhosis or aspartate transaminase (AST) to Platelet Ratio Index (APRI) score >2, thus none met the WHO guidelines for immediately starting treatment due to cirrhosis. APRI score was <2, but alanine transaminase (ALT) was deranged in 69.2% (18/26) of clinic attendees and they were advised to repeat tests in 3 months to ascertain if rise was transient or persistent. The remaining 8 people (30.7%) were advised to repeat blood tests in 12 months time, in accordance with 2015 WHO guideline (Table 3).

**Table 3:** Laboratory and clinical findings of participants with chronic hepatitis B.

	All HBsAg positive	Attended clinic
All	39	26 (100%)
Sex		
Male	8 (20.5%)	6 (23.1%)
Female	31 (78.5%)	20 (76.9%)
Age		
Under 30 years	9 (23%)	4 (15.4%)
30 years and older	30 (76.7%)	22 (84.6%)
APRI		
<1		25 (96.1%)
1–2		1 (3.8%)
> 2		0
ALT		
Normal		8 (30.8%)
Between x1 and x2 ULN		9 (34.6%)
> 2 times ULN		9 (34.6%)
HIV co-infection		1 (3.8%)
Clinical features of liver disease of cirrhosis		0

HBsAg = Hepatitis B Surface Antigen, APRI = Aspartate Transaminase (AST) to Platelet Ratio Index, ALT = alanine transaminase, ULN = Upper Limit Normal. Normal defined as AST < 19 IU/mL for women and <30 IU/mL for men.

Regarding knowledge, 90.4% (403 / 446) of participants were aware that hepatitis B could cause liver cancer, but only 11.7% (52 / 447) were able to answer all five correctly that that hepatitis B could be spread by of sexual contact, mother to child transmission and “needlestick injuries”, and that it could not be spread by holding hands or respiratory droplets. Most (96.9%, 432/ 446) healthcare workers were concerned about their risk of hepatitis B at work (Table 4).

**Table 4:** Questionnaire results.

	Number (%)
**Knowledge**	
**1**. “Can Hepatitis B causes liver cancer”–answers yes	403 (90.4%)
**2**. Correctly identifies all modes of transmission and does not identify non-correct modes of exposures *	52 (11.7%)
	Number (%) answering “agree”
**Attitudes**	
**3**. “I am at risk of having hepatitis B”	442 (99.1%)
**4**. “I would not want to be friends with someone with hepatitis B”	225 (50.4%)
**5**. “I am worried about my risk of hepatitis B at work”	432 (96.9%)
**Practices**	
**6**. “I have been vaccinated against hepatitis B”	66 (14.8%)
**7**. “I always have access to ‘sharps boxes’ to safely dispose of needles”	401 (89.9%)
**8**. “I know how to protect myself from hepatitis B at work”	320 (71.7%)
**9**. “I know what action I should take at work if I have a needle stick exposure at work”	303 (67.9%)

* Routes of transmission suggested were needle stick, mother to child, sexual exposure (correct answers) and holding hands and respiratory droplets (incorrect answers). Question posed was “Can hepatitis B be spread by” and prompts for routes followed by “yes / no / don’t know” options.

In the electronic questionnaire, 14.7% (66 / 447) participants reported having at least one dose of hepatitis B vaccine. Among this sub-set, 4.5% (3 people) had anti-HBs only (evidence of immunity secondary to vaccination), 4.5% (3/66) had HBsAg (i.e. chronic hepatitis B), 13.6% (9/66) had both anti-HBc and anti-HBs (consistent with immunity through natural infection), (10.6%, 7/66) had isolated anti-HBc and 68.2% (45/66) had no hepatitis B antigens detected. There were 11 participants who did not report having had the vaccine, but had a hepatitis B panel result consistent with previous vaccination.

## Discussion

We show a high prevalence of chronic hepatitis B in a population of healthcare workers in an urban West Africa setting (HBsAg positivity 8.7%). There are no official government estimates of prevalence, no routine surveillance for hepatitis B and no systematic sero-surveys. To our knowledge, this is the only recent systematic study of hepatitis B prevalence in a well-defined group in Sierra Leone. This prevalence is similar to that found in the Gambia in a large community based study (8.8% in the PROLIFICA study [11]) and to a study 2005 among pregnant women attending private clinics in Freetown (6.2% of 302 blood samples were positive for HBsAg in that study, from an unknown number of people targeted for testing [12]). The prevalence is lower than that reported in a 1997 community based study of primary school children in Freetown (18% of 66 children) [13], a 2012 hospital based cohort of febrile patients in a rural area of Sierra Leone (14% of 860 patients) [14] and a 2016 blood bank study in a rural area of Sierra Leone (15% of 326 blood donors) [15]. Our prevalence is also similar to a healthcare worker survey in South Africa (9.6% of 314 healthcare workers) [16].

In addition to the 8.7% of people with chronic hepatitis B in our study, a further 90 people (20%) had evidence of previous contact with HBV in the form of anti-HBc (40 people with anti-HBc and anti-HBs and 50 with “anti-HBc alone”). “Anti-HBc alone” is a common finding in other studies in populations with high HBsAg prevelance, for example one study of immigrants from sub-saharan Africa to Australia found this pattern in 20% of those screened [17], and a study of immigrants to Italy showed 15% anti-HBc alone [18]. This pattern most likely indicates previous exposure to HBV and clearance of virus, perhaps with an anti-HBs response which has waned over time. It is possible that for some of these participants the “anti-HBc alone” pattern may represent occult HBV infection [19]. Previous studies have shown that a small proportion of patients with “anti-HBc alone” have low levels of HBV DNA (1.7% in a large blood bank study in Korea [20] and 3% in the above study of immigrants to Australia [17]). The risk of occult HBV infection progressing to cirrhosis and chronic liver disease is unclear [5] [21]. In our study false-positive result as an explanation for “anti-HBc alone” is less likely as these tests were positive on both LFA and EIA. Acute infection is also unlikely as all participants were healthy and asymptomatic. We did not have access to HBV DNA testing to study this phenomenon “anti-HBc alone” further.

None of the 26 patients with chronic hepatitis B who attended for clinical assessment met the 2015 WHO criteria to recommend starting treatment on the basis of cirrhosis. However one person with HIV co-infection was referred to start tenofovir as part of combination therapy for HIV / HBV co-infection. This low prevalence of cirrhosis at the time of initial assessment is in keeping with the PROLIFICA study in The Gambia (4.4% of HBsAg positive participants recruited from community screening met European Association Study Liver Disease (EASL) criteria [11]), but lower than that reported in a cohort in Ethiopia (25% of HBsAg positive patients referred from healthcare providers to a central clinic for assessment met modified EASL criteria [22]). A previous economic modelling study about the potential cost of rolling out hepatitis B treatment, based on the Gambia data concluded that community testing and treatment for hepatitis B is economically feasible in the Gambia [23]. Our study suggests that these assumptions about a high HBsAg positivity rate but a low proportion of people requiring treatment would also be the case in Sierra Leone.

Voluntary hepatitis B screening was reasonably well accepted in our setting. It is difficult to be completely accurate about how many people were offered testing; it was not considered acceptable to do a census of all staff who were present at work during the study four weeks due to concerns about confidentiality of those who chose not to test. The hospital staff register does not take into account study leave, annual leave or other causes of absenteeism. Between 46–72% of doctors and nurses at Connaught hospital accepted voluntary testing based either on the total number of staff posted to Connaught Hospital (46% tested) or the numbers counted during ward mobilisation (72% tested). At 34 Military Hospital, 57% of doctors and nurses accepted testing. This is similar to coverage of community screening for HBV in the Gambia (69%)[11] and to community HIV screening in Uganda (63%) [24], but lower than door to door HIV screening a multicentre Southern Africa setting (82%) [25]. Screen and treat is a pressing issue for hepatitis B management in sub-Saharan Africa, especially as efforts are scaled up to meet elimination targets. This study shows that following sensitisation, healthcare workers in Sierra Leone are willing to undergo confidential workplace-based hepatitis B screening, although is does not necessarily follow that this would be replicated in the general population.

Generally participants had some knowledge (90% knew that hepatitis could lead to liver cancer) but only 12% of participants could correctly identify all ways that hepatitis was transmitted. This may be due in part to infection prevention and control training that has focused primarily on viral haemorrhagic fevers, and teaching about avoiding respiratory droplet exposure. Half of healthcare workers surveyed reported that they wouldn’t wish to be friends with someone with hepatitis B, suggesting high levels of stigma towards people with hepatitis B. There are high levels of concern about occupational exposure to hepatitis B and almost universal perception by staff that they are at risk. There are no clear estimates about the amount of hepatitis B that is contracted through occupational exposure compared to other routes [7]. In a high prevalence setting like Sierra Leone, where many people contract chronic hepatitis B in early infancy it is not clear whether healthcare workers are at higher risk than the general population for hepatitis B. High levels of anxiety about hepatitis B and other blood borne virus transmission may lead to healthcare worker losses due to fear of transmission of viruses, or to discrimination against patients who are perceived as posing a risk of transmission to the healthcare worker [7] [26]. Further education of healthcare staff about occupational risk of hepatitis B, in conjunction with adequate access to personal protective equipment (PPE) and “sharps boxes” at work would be beneficial.

Only 4.5% of those reporting having had at least one dose of hepatitis B vaccine had evidence of an anti-HBs response. It is difficult to know how to interpret this finding, especially as the stated LOD for the anti-HBs LFA was 30 IU/mL and it is thought that over 10 IU/mL is protective. It may be that staff had a low level of antibody that is still protective but undetectable using this assay, or it could be that insufficient vaccine doses were given or that immune responses have waned over time. Hepatitis B vaccination is not generally provided by employers for healthcare workers and is not provided by the government for adults.

There are some limitations to the present study. As already mentioned it is difficult to be certain of the coverage and the exact number of staff invited to participate. In line with 2015 WHO recommendations we used APRI with a cut-off value of 2 to assess for presence cirrhosis [4]. There is some discussion in the literature about whether APRI is sufficiently sensitive to assess cirrhosis, particularly in an African setting [27] [22]. We did not have access to alternative modalities (hepatic elastography) to assess liver fibrosis and compare to APRI score. There are no validated Knowledge Attitudes and Practices surveys for hepatitis B, which limits the inference that can be drawn from our short survey [28]. Many of the limitations are as a result of the resource constraints within Sierra Leone. We did not have resources to perform EIA testing on all negative samples, despite the EIA being a more sensitive and specific test with a lower stated LOD (2 IU/mL vs 5 IU/mL) for HBsAg. All positive samples by LFA were confirmed by EIA testing with no false-positives. There were no false-negative HBsAg results among the subset with anti-HBc alone on LFA—this group was potentially high risk for low level HBsAg positivity. Hepatitis B virus DNA testing for viral load detection is unaffordable, and hepatic elastography is not, to our knowledge, available in Sierra Leone. There is no ability to genotype samples. Public healthcare resources are extremely limited and high out of pocket costs restricts access to long-term follow-up and treatment. We do not have follow-up information for patients after their initial assessment (which was free for the patient) and it is possible that many patients were unable to afford the recommended monitoring strategy following the initial free consultation. Others have described similar challenges in other countries in sub-Saharan Africa[5].

We have shown a high prevalence of hepatitis B in healthcare workers in Sierra Leone, in keeping with the known high prevalence in nearby West African countries. There is also a high prevalence of anti-HBc suggesting that many people who do not currently have proven chronic infection have had contact with hepatitis B previously. Our questionnaire shows a high level of concern about hepatitis B exposure among healthcare workers. Encouragingly, there is a low rate of complications at present among this cohort of patients. However, ongoing monitoring and potential future treatment costs are beyond the means of most patients to pay privately. We were encouraged by the call of the current Minister of Health for Sierra Leone for “urgent action” to be taken to prevent more infections and minimise their health consequences [15]. In order to achieve the 2030 goal of elimination of viral hepatitis, this action must take the form of strengthening routine EPI immunisation programmes, rolling out birth-dose vaccination, catch-up vaccination for high risk adults (including healthcare workers) and working towards a national screen and treat programme, with healthcare workers as a priority group.

## Supporting information

S1 Appendix Manufacturer reported sensitivity, specificity and limits of detection of hepatitis B serology tests used. (DOCX)

S2 Appendix Question items in electronic questionnaire. (PDF)

S3 Appendix Anonymised demographic, serological, clinical and questionnaire results. (XLSX)
