# Supplementary material for: Cross sectional study of chronic hepatitis B prevalence among healthcare workers in an urban setting, Sierra Leone
Source: PLoS One. 2018 Aug 10;13(8):e0201820. doi: 10.1371/journal.pone.0201820 (PMC6086405; doi:10.1371/journal.pone.0201820)
Supplement: S1 Appendix — (DOCX) [file pone.0201820.s001.docx]

The manufacturer report specificity, sensitivity and limits of detection for the hepatitis B serology panel as follows;

Shanghai KHB Multi-HBV Markers Colloidal Gold Lateral Flow Assay (LFA)

HBsAg, 99.5% specific, 99.1% sensitive, 5IU/mL LOD; anti-HBs, 99.5% specific, 99.4% sensitive, 30mIU/ML LOD; anti-HBc 99.7% specific, 90.8% sensitivity, no LOD stated; anti-HBe, 98.2% specific, 99.3% specific, no LOD stated, HBeAg 94.4% specific, 100% sensitive, no LOD.

Wantai Bio-Pharm ELISA kits (each epitope is a separate kit)

HBsAg, 100% sensitive, 99.8% specific, 0.2IU/mL LOD; anti-HBs 100% sensitive, 100% specific, 10 IU/mL LOD; anti-HBc, 100% specific, 100% sensitive, 1 IU/mL LOD; HBeAg, 100% sensitive, 99.9% specific, 1 IU/mL LOD; anti-HBe, 100% sensitive, 99.2% specific, 1 IU/mL LOD.
