# Supplementary material for: Cross sectional study of chronic hepatitis B prevalence among healthcare workers in an urban setting, Sierra Leone
Source: PLoS One. 2018 Aug 10;13(8):e0201820. doi: 10.1371/journal.pone.0201820 (PMC6086405; doi:10.1371/journal.pone.0201820)
Supplement: S2 Appendix — (PDF) [file pone.0201820.s002.pdf]

Hepatitis B prevalence in healthcare workers in Freetown, Sierra Leone

This is a short questionnaire to find out about healthcare workers knowledge, attitudes and practices regarding hepatitis B. It should take less than 5 minutes to complete. Thank you.

CODE (WORD)

CODE (NUMBER)

ABOUT YOU

Gender (please circle)

Age category (years)

What is your profession?

KNOWLEDGE ABOUT HEPATITIS B

1. Can Hepatitis B cause liver cancer?

2. Can hepatitis B be spread by:

a. Mother to child transmission

b. Sexual contact

c. By sneezing or respiratory droplets

d. Through unsterilized surgical equipment and "needle stick" injuries.

e. Holding hands

ATTITUDES ABOUT HEPATITIS B

3. I am at risk of having hepatitis B

4. I would not want to be friends with someone who has Hepatitis B

5. I am worried about my risk of Hepatitis B at work

PROFESSIONAL PRACTICES REGARDING HEPATITIS B

6a. I have been vaccinated for Hepatitis B

6b. How many doses of Hepatitis B vaccine did you have?

7. I always have access to sharp box to safely dispose of needles

8. I know how to protect myself from Hepatitis B at work

9. I know what action I should take at work if I have a needle stick exposure at work
